# Rheumatological Findings in Candidates for Valvular Heart Surgery

**DOI:** 10.5402/2012/927923

**Published:** 2012-10-31

**Authors:** Mohammad Bagher Owlia, Seyed Jalil Mirhosseini, Nafiseh Naderi, Seyed Mohammad Yousof Mostafavi Pour Manshadi, Sadegh Ali Hassan Sayegh

**Affiliations:** ^1^Department of Medicine, Shahid Sadoughi University of Medical Sciences and Health Services, Yazd, Iran; ^2^Department of Cardiac Surgery, Afshar Hospital, Shahid Sadoughi University of Medical Sciences and Health Services, Yazd, Iran; ^3^Department of Medicine, Ali ben Abitaleb Medical College, Islamic Azad University, Yazd, Iran; ^4^Yazd Cardiovascular Research Center, Afshar Hospital, Shahid Sadoughi University of Medical Sciences and Health Services, Yazd, Iran

## Abstract

*Background and Objectives*. Valvular heart diseases are among the frequent causes of cardiac surgery. Some patients have a well-known rheumatic condition. Heart valves are fragile connective tissues which are vulnerable to any systemic autoimmune diseases. This study was designed to evaluate the frequency of rheumatological background in patients candidate for valvular heart surgery in Afshar Cardiovascular Center, Yazd, Iran. *Methods*. One hundred and twenty (120) patients candidate for valvular heart surgery were selected for this study. Careful history and physical examination were undertaken from rheumatological stand points. The most sensitive screening serologic tests were also assayed. *Results*. The result of this study showed that 53.3% were male and 46.6% were female with mean age of 48.18 ± 17.65 years old. 45.8% of the patients had history of nonmechanical joint disease, 14.2% had history of rheumatological conditions in their family, and 30% had history of constitutional symptoms. 29.8% had positive joint dysfunction findings in their physical examination while 25.8% had anemia of chronic disease. Positive Rheumatoid factor (RF), anticyclic citrullinated peptide (CCP, ACPA), C-reactive protein (CRP), antinuclear antibody (ANA), abnormal urine and elevated erythrocyte sedimentation rate (ESR) were 34, 2.5, 26.7, 4.2, 5, and 36.7%, respectively. Antineutrophil cytoplasmic antibody (ANCA) and antiphospholipid (APL) were positive in a few cases. *Conclusion*. The findings of this study show immunologic bases for most patients with valvular heart diseases candidate for surgery. Undifferentiated connective tissue diseases may play an important role in the pathophysiology of valvular damage.

## 1. Introduction

Valvular heart diseases (VHD) surround a number of common cardiovascular disorders that account for 15% of all cardiac surgical procedures in the world. Patients with VHD require intervention for improvement, relief, or valve replacement. One of the most important pathogeneses of coronary artery stenosis and VHD is inflammatory response of immune system. Prevalence and incidence of cardiovascular diseases resulting from rheumatologic disorders are increasing, however, advanced treatments which are now available [1–3]. Rheumatic diseases can be assumed by taking a clinical history and conducting thorough physical examination. Laboratory and radiographic investigations can help to diagnose more accurately; however, clinical skills and expertise are an essential factor in diagnosing undifferentiated connective tissue diseases. The frequent complaints of rheumatologic disorders include pain and swelling of joints and signs of loss of function in different organs [4, 5]. This study was designed to evaluate the frequency of rheumatological manifestations and serologic evaluation in patients candidate for valvular surgery in Afshar Cardiovascular Center, Yazd, Iran.

## 2. Material and Methods 

Our cross-sectional study was approved by the ethics committee of our university. After receiving the written consent from the patients, they had undergone valvular heart surgery from May, 2010 to November, 2011 in Afshar Cardiovascular Center, Yazd, Iran. All operations were performed by a consultant surgeon. One hundred and twenty patients candidate for valvular heart surgery were selected for this study. History taking and thorough physical examination were performed for all of them. Preoperative tests such as C-reactive protein (CRP), erythrocyte sedimentation rate (ESR), complete blood count (CBC), urine analysis (UA), antinuclear antibody (ANA), antiphospholipid (APL) (IgM & IgG), antineutrophil cytoplasmic antibody (ANCA), antistreptolysin-O (ASO), and rheumatoid factor (RF) were performed on patients' samples. Pathological and echocardiography findings along with the results of serologic and rheumatologic tests were saved in a questionnaire. Data were analyzed using SPSS version 15 software. ANOVA, chi-square, and *t*-test were used for quantitative and qualitative variables.

## 3. Results

One hundred and twenty (120) patients candidate for valvular heart surgery were selected for this study. Their average age was 48.18 ± 17.65 years. Of these cases, 64 cases (53.3%) were males and 56 cases (46.6%) were females. The positive medical history of patients is shown in Table 1.

Distribution of clinical findings in participants was important because 17 cases (14.2%) had familial history of rheumatism and 55 cases (45.8%) had joints dysfunction. The positive clinical findings in our patients are presented in Table 2.

White blood cells counts were divided into two groups (>4000 and <4000); of all the patients, 3 (2.5%) were leucopenia, 31 (25.8%) had anemia with hemoglobin concentration <11, and 18 (15%) had platelet count <140000 as thrombocytopenia. Of all the patients, 79 (65.8%) had negative rheumatoid factor (RF), while 41 (34.2%) had positive RF; however, of all the cases with positive RF, 28 cases (23.3%) had one positive RF, and 13 cases (10.8%) had double positive RF. Of the 120 patients, 88 (73.3%) had negative CRP, while 32 (26.7%) had positive CRP; however, 26 cases (21.7%), 5 cases (4.2%), and 1 case (0.8%) had single, double, and triple positive CRP, respectively. Patients with positive results (higher than one) for some tests such as ANA, ANCA-C, and ANCA-P were 5 cases (4.2%), 1 case (0.8%), and 3 cases (2.5%) respectively. Of all the patients, 2 cases (1.7%) had APL-IgM, while two other cases (1.7%) had APL-IgG > 10, respectively. We had 3 cases (2.5%), with anti CCP > 30 (positive result), and 13 cases (10.8%) with ASO > 250 (positive result). Pathologic and echocardiography findings of all patients are presented in Tables 3 and 4, respectively.

## 4. Discussion

Heart and rheumatism are to words closely related together since centuries. Both patients and physicians are concerned about involvement of heart in rheumatic diseases, because the immunologic damage to the heart is usually irreversible. This is a “truth” not a “myth.” Cardiac tissues especially leaflet are extremely vulnerable to the process of inflammation and autoimmunity. Heart was the major concern in the era of discovery of acute rheumatic fever in 1950s, when little was known about spectrum of highly protean rheumatic diseases and the serologic tools for differentiation of apparently similar conditions were minimal [6]. Heart and joints are the most moving organs in the body and authors suppose that this common feature may be a factor for this excess vulnerability. All layers of heart are susceptible to immune injury and we believe that “the more intensity of systemic inflammation, the more probability of heart to be involved.” The examples are systemic lupus erythematosus and acute rheumatic fever or even a frank sepsis. Lower grades of smoldering involvements can be seen in almost all other rheumatic diseases [7]. Accelerated coronary artery disease is coming to be a concern in conditions like rheumatoid arthritis [8]. Patients under cardiac valve surgery, rather than congenial diseases, usually have hidden inflammatory background, and these backgrounds can be known as rheumatologic condition such as rheumatoid arthritis (RA), lupus, antiphospholipids syndrome, or be some kinds of undifferentiated connective tissue diseases. 

In a study by Horstkotte et al. [9], about 77% of 1051 patients who were waiting for operation on mitral valve stenosis had some features of rheumatism. The results of the above study were in accordance with those of this study, and its results confirm the necessity of this study. In the study of Penmetcha et al. [10], severe mitral valve regurgitation in a case of scleroderma/systemic lupus overlap was reported. And in a report by Leff et al. [11], acute aortic insufficiency in a 17-year-old patient with Wegener's granulomatosis was reported.

In the study of Lee et al. [12], aortic valve involvement in Behçet's disease was investigated. In the study of Evangelopoulos et al. [13], relationship between mitral valve prolapse and systemic lupus was observed. Mitral valve prolapse was diagnosed in 19 out of 87 patients with lupus. Also, anticardiolipin antibodies were higher in lupus patients with mitral prolapse [13].

In the study of Maksimowicz-McKinnon and Mandell [14], cardiac valvular diseases were investigated in patients with systemic autoimmune disorders of RA, lupus, antiphospholipid syndrome, seronegative arthropathies, systemic vasculitis, and scleroderma, and different valvular disorders were shown [14]. In the study of Doria et al. [15], cardiac involvement was investigated in lupus, and it was observed that pericarditis was the most common cardiac involvement in the lupus. Valve damage, myocarditis, and coronary vascular involvements were among other cardiac involvements that was reported in the lupus [15]. In the study of Tenedios et al. [16], cardiac involvement in antiphospholipid syndrome was investigated; cardiac manifestation of antiphospholipid, valve abnormalities such as vegetation and valve thickening were reported. Also, atherosclerosis and infracted myocardium, intracardiac thrombus, ventricle functional disorders, and pulmonary hypertension were reported in lupus [16]. 

 In a study by Atzeni et al. [17], Behçet's disease and its cardiac involvement were investigated. Cardiac manifestations in 7% to 46% of the patients and mortality of 20% were reported. Endocarditis, myocarditis, pericarditis, acute myocardial infarction, ventricular thromboses, congestive cardiomyopathy, and functional valve disorder were also reported in these patients [17]. 

 In the study of Kamiński et al. [18], 50 patients diagnosed of RA were studied according to the American College of Rheumatology Criteria. The results indicated that RA leads to valve degeneration, and these damages are related to atrial and ventricular hypertrophy [18]. In a study by Voskuyl, pericarditis, cardiomyopathy, myocarditis, coronary vasculitis, arrhythmia, and valve diseases were observed in a group of RA patients. Congestive heart failure and ischemic heart disease (IHD) were also more prevalent [19]. The results of a study conducted by Guilherme et al. [20] indicated that in 30% to 45% of patients, serious complication of rheumatic fever and chronic valve damages is evident.

In one study conducted by Santiago et al., valvular diseases were investigated in lupus and valvulopathy in 17 patients (15%), pulmonary hypertension in 7 patients (6.2%), and pericardial effusion in 2 patients (1.8%) were observed [21]. 

In a study by Gabrielli et al. [22], valvular involvement in systemic lupus and antiphospholipid syndrome was investigated; valve damages were observed in 15 patients with systemic lupus (38%). These abnormalities were mitral valve thickening or vegetation, mitral valve prolapse, aortic valve vegetation, mitral valve regurgitation and aortic, tricuspid, and mitral valve stenosis. In the systemic lupus, high level of anticardiolipin antibodies was reported in 73% of patients with valve damages and in 67% of patients without valve damages [22]. Anticardiolipin antibodies were also reported in 73% of patients with valve damages and 62% of patients without valve damages. Thus, the result was in accordance with that of the present study. In a study by Kitas et al., cardiac involvement was studied in patients with rheumatologic disorders and he showed some degrees of valve involvement in RA. Myocarditis was found in 30% of the autopsied patients. There was ischemic heart disease (IHD) in 50% of the RA patients [4]. 

 In a study by Neumann et al. [23], cardiac involvement was studied in 49 patients with Churg-Strauss syndrome. Twenty-two patients had clinical evidences of cardiac involvement. Negative ANCA and high eosinophil count were reported in the patients with cardiac involvement. Functional disorder of left ventricle (50%), valve insufficiency (73%), and pericardial effusion (41%) were the most common findings in these patients [23]. 

In a study by Beckhauser et al., valve involvements in RA patients were investigated. Twenty-eight RA patients (15.2%) were recognized with valve disease. Valve damages were more common in patients whose disease was more than 15 years and the aortic valve was the most common. There was no relationship between valve involvement and gender, age, and exposure to tobacco, positive rheumatoid factor, presence of ANA, rheumatoid nodules, and anticardiolipin antibodies [24]. 

In this study, high frequency of positive results for RF, ANA, and markers of inflammation shows that inflammation in general and autoimmunity per se may have a crucial role in most of valvular diseases leading to surgical repair.

## 5. Conclusion

Various rheumatologic conditions may lead to valvular involvements necessitating heart surgery in some instances. Basic mechanism in valvular heart diseases is possibly the same or at least similar as the pathophysiology of autoimmune diseases. The role of rheumatic conditions other than acute rheumatic fever in the etiology of valvular heart diseases may probably be underemphasized over decades. 

This project is the result of medical thesis of Dr. N. Naderi practicing as general practitioner. 

## Figures and Tables

**Table 1:** Positive medical history in the studied patients.

Positive medical history	Number	Percentage
Familial history of rheumatism	17	14.2
Joint dysfunction	55	45.8
Recurrent infection	16	13.3
Skin or mucous disease	16	13.3
Neurologic disease	23	19.2
Eye disease	13	10.8
Ear disease	11	9.2
History of chronic pulmonary disease	23	19.2
History of MI or vein thrombosis	15	12.5
Pericarditis or arrhythmia	14	11.7
Chronic gastric disease	9	7.4
Abortion	10	8.3
Renal disease	18	15
Hypothyroid	5	4.1
Diabetic mellitus	30	25

**Table 2:** Positive clinical findings in our patients.

Positive clinical findings	Number	Percentage
Dysfunction of peripheral and axial joints	35	29.8
Skin, hair, and nail disease	10	8.3
Mucous disease	10	8.3
Pulmonary or cardiovascular disease	100	83.3
Lymphatic gland disease	6	5
Thyroid disease	7	5.8
Abdominal disease	7	5.8
Neurologic disease	10	8.3

**Table 3:** Pathologic results from heart valve tissues in the studied patients.

Variables	Number	Percentage
Acute or chronic inflammation	36	30
Rheumatism	33	26.6
Degeneration of valves	49	40.8
Endocarditic	2	1.6

**Table 4:** Echocardiographic findings in our cases.

Variables	Mild (*n* (%))	Moderate (*n* (%))	Severe (*n* (%))
Mitral valve insufficiency	12 (10)	22 (18.3)	17 (14.2)
Mitral stenosis	3 (2.5)	19 (15.8)	35 (29.2)
Aortic valve insufficiency	12 (10)	24 (20)	27 (22.5)
Aortic stenosis	2 (1.7)	4 (3.3)	9 (7.5)
Tricuspid valve insufficiency	13 (10.8)	5 (4.2)	7 (5.8)
Tricuspid stenosis	0 (0)	0 (0)	2 (1.7)
Pulmonary valve insufficiency	2 (1.7)	0 (0)	1 (0.8)
Pulmonary stenosis	0 (0)	0 (0)	0 (0)

## References

[1] Nishimura RA, Carabello BA, Faxon DP (2008). ACC/AHA 2008 guideline update on valvular heart disease: focused update on infective endocarditis: a report of the American college of cardiology/American heart association task force on practice guidelines endorsed by the society of cardiovascular anesthesiologists, society for cardiovascular angiography and interventions, and society of thoracic surgeons. *Journal of the American College of Cardiology*.

[2] Bonow RO, Carabello BA, Chatterjee K (2008). 2008 focused update incorporated into the ACC/AHA 2006 guidelines for the management of patients with valvular heart disease: a report of the American college of cardiology/American heart association task force on practice guidelines (writing committee to revise the 1998 guidelines for the management of patients with valvular heart disease): endorsed by the society of cardiovascular anesthesiologists, society for cardiovascular angiography and interventions, and society of thoracic surgeons. *Circulation*.

[3] Otto CM, Bonow RO, Zipes DP, Libby P, Bonow RO, Braunwald E (2007). Valvular heart disease. *Braunwald's Heart Disease: A Textbook of Cardiovascular Medicine*.

[4] Kitas G, Banks MJ, Bacon PA (2001). Cardiac involvement in rheumatoid disease. *Clinical Medicine*.

[5] Giles JT, Bartlett SJ, Andersen R, Thompson R, Fontaine KR, Bathon JM (2008). Association of body fat with C-reactive protein in rheumatoid arthritis. *Arthritis and Rheumatism*.

[6] Owlia MB (2011). Acute rheumatic fever, an acute or a chronic joint disease?. *Journal of Shahid Sadoughi University of Medical Sciences*.

[7] Owlia MB (2006). Clinical spectrum of connective tissue disorders. *Journal, Indian Academy of Clinical Medicine*.

[8] Owlia MB, Mehrpoor G (2012). Behcet's disease: new concepts in cardiovascular involvements and future direction for treatment. *ISRN Pharmacology*.

[9] Horstkotte D, Niehues R, Strauer BE (1991). Pathomorphological aspects, aetiology and natural history of acquired mitral valve stenosis. *European Heart Journal*.

[10] Penmetcha M, Rosenbush SW, Harris CA (1996). Cardiac valvular disease in scleroderma and systemic lupus erythematosus/scleroderma overlap associated with antiphospholipid antibodies. *Journal of Rheumatology*.

[11] Leff RD, Hellman RN, Mullany CJ (1999). Acute aortic insufficiency associated with Wegener granulomatosis. *Mayo Clinic Proceedings*.

[12] Lee CW, Lee J, Lee WK (2002). Aortic valve involvement in behçet’s disease. A clinical study of 9 patients. *Korean Journal of Internal Medicine*.

[13] Evangelopoulos ME, Alevizaki M, Toumanidis S (2003). Mitral valve prolapse in systemic lupus erythematosus patients: clinical and immunological aspects. *Lupus*.

[14] Maksimowicz-McKinnon K, Mandell BF (2004). Understanding valvular heart disease in patients with systemic autoimmune diseases. *Cleveland Clinic Journal of Medicine*.

[15] Doria A, Iaccarino L, Sarzi-Puttini P, Atzeni F, Turriel M, Petri M (2005). Cardiac involvement in systemic lupus erythematosus. *Lupus*.

[16] Tenedios F, Erkan D, Lockshin MD (2005). Cardiac involvement in the antiphospholipid syndrome. *Lupus*.

[17] Atzeni F, Sarzi-Puttini P, Doria A, Boiardi L, Pipitone N, Salvarani C (2005). Behçet’s disease and cardiovascular involvement. *Lupus*.

[18] Kamiński G, Makowski K, Dziuk M (2005). Degenerative valvular and left ventricle structural changes in echocardiography in patients with rheumatoid arthritis. *Polski Merkuriusz Lekarski*.

[19] Voskuyl AE (2006). The heart and cardiovascular manifestations in rheumatoid arthritis. *Rheumatology*.

[20] Guilherme L, Köhler KF, Kalil J (2011). Rheumatic heart disease. Mediation by complex immune events. *Advances in Clinical Chemistry*.

[21] Santiago MB, Dourado SMM, Silva NO (2011). Valvular heart disease in systemic lupus erythematosus and Jaccoud’s arthropathy. *Rheumatology International*.

[22] Gabrielli F, Alcini E, Di Prima MA, Mazzacurati G, Masala C (1995). Cardiac valve involvement in systemic lupus erythematosus and primary antiphospholipid syndrome: lack of correlation with antiphospholipid antibodies. *International Journal of Cardiology*.

[23] Neumann T, Manger B, Schmid M (2009). Cardiac involvement in churg-strauss syndrome: impact of endomyocarditis. *Medicine*.

[24] Beckhauser AP, Vallin L, Burkievcz CJ, Perreto S, Silva MB, Skare TL (2009). Valvular involvement in patients with rheumatoid arthritis. *Acta Reumatologica Portuguesa*.

