# Corrigendum to “Rheumatological Findings in Candidates for Valvular Heart Surgery”

**DOI:** 10.1155/2017/4704168

**Published:** 2017-11-27

**Authors:** Mohammad Bagher Owlia, Seyed Jalil Mirhosseini, Nafiseh Naderi, Seyed Mohammad Yousof Mostafavi Pour Manshadi, Sadeq Ali-Hasan-Al-Saegh

**Affiliations:** ^1^Department of Medicine, Shahid Sadoughi University of Medical Sciences and Health Services, Yazd, Iran; ^2^Department of Cardiac Surgery, Afshar Hospital, Shahid Sadoughi University of Medical Sciences and Health Services, Yazd, Iran; ^3^Department of Medicine, Ali ben Abitaleb Medical College, Islamic Azad University, Yazd, Iran; ^4^Yazd Cardiovascular Research Center, Afshar Hospital, Shahid Sadoughi University of Medical Sciences and Health Services, Yazd, Iran

In the article titled “Rheumatological Findings in Candidates for Valvular Heart Surgery” [1], the name of the last author was given incorrectly as Sadegh Ali Hassan Sayegh. The author's name should have been written as Sadeq Ali-Hasan-Al-Saegh. The revised authors' list is shown above. The published spelling was the Persian transliteration, whereas the corrected spelling is the Arabic transliteration.
